# A New Needle for Sutures

**Published:** 1860-10

**Authors:** 


### A NEW NEEDLE FOR SUTURES.

---

In the October number of the *Nashville Journal of Medicine and Surgery*, for 1860, Prof. Paul F. Eve has a description of a needle of his invention designed chiefly for metallic sutures. The subjoined extract will indicate what is claimed for it by Prof. Eve.; and perhaps will give as good an idea of the instrument as can be conveyed without the cut that accompanies its description as originally published:

"The needle is mounted on a fixed handle, and is therefore under the control of the operator. It is slightly curved, and has a lancet-like point and shoulders, to facilitate its passage through the soft parts. The novelty of its construction is the *canula* at the curvature through which the ligature, metallic or otherwise, is passed, and this may vary from one-half to an inch or two in length. I have had three sizes made, the smallest for silver wire, and the larger for lead. I have just sent an order to Mr. Tiemann, for one very delicate, more curved, and with a longer handle, for a case of staphyloraphy now under treatment for operation.

The needle, armed with the ligature deposited in the canula, is made to transfix the flaps gently held together; the wire now pushed through the canulated portion is seized and held by the fingers while the needle itself is withdrawn. The sutures are thus properly placed, with great ease and but little pain, and the wound is ready for dressing. My plan is not to cut off the extremities of the ligatures close to the knot, but simply to twist the wire to close the wound, then lay its two extremities down on the integuments, and there secure them by adhesive strips during the healing process. In this way the wound is not liable to be injured by their sharp cut ends, and the same ligature may be repeatedly used.

Obnoxious to the single objection of size, for I have long advocated small suture needles, knowing well how patients suffer in stitching the flesh; the one here described is certainly quite simple, inexpensive, (costing but $2 apiece,) is of easy, accurate, and of general, if not universal applicability. For instance, in vesico-vaginal fistula, the needles now in use are not only brought through, but out of the edges surrounding the opening, a very difficult and painful step in the operation, and then a silk ligature is introduced, to be supplanted by that of silver; whereas by the new needle here proposed, the wire is immediately and readily applied by simply transfixing the soft parts and drawing it from the canula. Again, by reversing its action, that is, by first transfixing the flaps, then placing a waxed thread into the opening of the canula, near the point, in now withdrawing the needle, the ligature is deposited, and by repeating this movement, the continued suture is made. The cadaver may thus be rapidly, and with great ease, stitched up after a post mortem examination, by simply thrusting this sharp instrument through the edges of the incisions, and arming the canula with a small cord at its point.

